# Toxicological effects of bioactive peptide fractions obtained from *Bothrops jararaca* snake venom on the structure and function of mouse seminiferous epithelium

**DOI:** 10.1590/1678-9199-JVATITD-2020-0007

**Published:** 2020-06-22

**Authors:** Carlos Alberto-Silva, Celline Sampaio Franzin, Joyce Meire Gilio, Rodrigo Simão Bonfim, Samyr Machado Querobino

**Affiliations:** 1 Natural and Humanities Sciences Center, Experimental Morphophysiology Laboratory, Federal University of ABC (UFABC), São Bernardo do Campo, SP, Brazil.; 2 Department of Biophysics, Federal University of São Paulo (Unifesp), São Paulo, SP, Brazil.; 3 Center for Health and Biological Sciences, Minas Gerais State University (UEMG), Passos, MG, Brazil.

**Keywords:** Bothrops jararaca, Snake venom, Bradykinin-potentiating peptides, Testis, Seminiferous epithelium, 15P-1 Sertoli cells, Spermatogenesis

## Abstract

**Background:**

Pathogenesis of *Bothrops* envenomations is complex and despite numerous studies on the effects of this snake venom on various biological systems, relatively little is known about such effects on the male reproductive system. In the present study, the toxicological outcomes of the low molecular weight fraction (LMWF) of *B. jararaca* snake venom - containing a range of bioactive peptides - were investigated on the dynamics and structure of the seminiferous epithelium and 15P-1 Sertoli cells viability.

**Methods:**

LMWF (5 µg/dose per testis) venom was administered in male Swiss mice by intratesticular (i.t.) injection. Seven days after this procedure, the testes were collected for morphological and morphometric evaluation, distribution of claudin-1 in the seminiferous epithelium by immunohistochemical analyses of testes, and the nitric oxide (NO) levels were evaluated in the total extract of the testis protein. In addition, the toxicological effects of LMWF and crude venom (CV) were analyzed on the 15P-1 Sertoli cell culture.

**Results:**

LMWF induced changes in the structure and function of the seminiferous epithelium without altering claudin-1 distribution. LMWF effects were characterized especially by lost cells in the adluminal compartment of epithelium (spermatocytes in pachytene, preleptotene spermatocytes, zygotene spermatocytes, and round spermatid) and different stages of the seminiferous epithelium cycle. LMWF also increased the NO levels in the total extract of the testis protein and was not cytotoxic in concentrations and time tested in the present study. However, CV showed cytotoxicity at 10 μg/mL from 6 to 48 h of treatment.

**Conclusions:**

The major finding of the present study was that the LMWF inhibited spermatozoa production; principally in the spermiogenesis stage without altering claudin-1 distribution in the basal compartment. Moreover, NO increased by LMWF induce open of complexes junctions and release the germ cells of the adluminal compartment to the seminiferous tubule.

## Background

Snakebite envenoming constitutes a serious public health problem according to the World Health Organization (WHO). Approximately 81,000 to 138,000 people die annually as a result of snakebite, and about three times as many amputations and other permanent disabilities are caused by snake envenomation every year [1]. 

*Bothrops jararaca* snake venom constitutes a complex mixture of proteins, such as phospholipase A2, serine proteinase, metalloproteinase, cysteine-rich secretory protein, lectin-like protein, C-type lectin, L-amino acid oxidase, and disintegrins [2]. Besides them, a variety of pharmacologically active peptides have been identified, such as proline-rich oligopeptides, also known as bradykinin potentiating peptides (BPPs) from the low molecular weight fraction (LMWF) of the venom [3]. Typically, BPPs contain 5 to 13 amino acid residues with a pyroglutamyl residue (<E) at the N-terminus and a proline residue at the C-terminus. BPPs longer than seven amino acids share similar features, including a high content of proline residues and the tripeptide sequence Ile-Pro-Pro at the C-terminus [4-7]. 

The pathogenesis of systemic effects of *Bothrops* envenomation is complex, involving both the direct action of venom components on the tissues and the release of various endogenous mediators [8] that provoke prominent local tissue damage and systemic disturbances such as hemorrhage, coagulopathies, cardiovascular shock and renal alteration [8-10]. Despite extensive studies on the effects of snake venom on different biological systems, relatively little is known about their effects on male reproductive system. 

Reprotoxin, a protein complex toxin from *Daboia russelii* venom (from western India), was the first toxin isolated from snake venom to be classified as toxic to the reproductive system. It induced atrophy in the Leydig cells, the Sertoli cells, and the seminiferous tubules of mouse testis and hemorrhage in the peritoneal cavity of experimental mice [11]. Another study indicated that *Crotalus durissus* ssp. rattlesnake venom (25 μg/kg of body weight) affected chromatin condensation and increased the number of sperm with abnormal morphology and the sperm count in sexually mature male CF-1 mice [12]. 

The effects of compounds from *B. jararaca* snake venom on the male reproductive system, particularly on spermatogenesis, have also been studied by our group. BPP-10c (<ENWPHQIPP), a peptide from *B. jararaca* snake venom, was described as a potent selective C-domain inhibitor of angiotensin-converting enzyme (sACE). However, this peptide showed inhibitory effects on the spermiogenesis without affecting the permeability of blood-testis barrier (BTB) and the distribution of claudin-1 in the male Swiss mice [13]. Additionally, the effects of different synthetic peptides [BPP-10c (<ENWPHQIPP), BPP-11e (<EARPPHPPIPP), BPP-AP (<EARPPHPPIPPAP)] and captopril were evaluated after injection into the testicular parenchyma (120 nmol/dose per testis) [14]. BPP-10c and BPP-AP, for example, showed an intense disruption of the epithelium and high degree of seminiferous tubule degeneration. Curiously, no morphological or morphometric alterations were observed in animals treated with captopril or BPP-11e [14]. These data have suggested that the alterations in the structure and function of the seminiferous epithelium in mice are dependent on their primary molecular structure and cannot be generalized for other BPPs [14]. In the current study, we investigated the toxicological effects of LMWF obtained from *B. jararaca* snake venom, on the dynamic and structure of the seminiferous epithelium in mice following intratesticular (i.t.) injection. 

## Methods

### Chemicals

All chemicals used in the present study were of analytical reagent grade (purity higher than 95%) and purchased from Calbiochem-Novabiochem Corporation (USA), Gibco BRL (USA), Fluka Chemical Corp. (Switzerland) or Sigma-Aldrich Corporation (USA).

### Animals

Male Swiss mice aged between 7 to 8 weeks (body weight from 30 to 35 g) were obtained from the Nuclear and Energy Research Institute (IPEN/CNEN-SP). Six animals were housed per cage, in 12-h light/dark cycle, constant exhaust ventilation (Alesco^®^, Brazil) and receiving standardized mouse chow (Nuvital Nutrientes Ltda, Brazil) *ad libitum*. The use of animals for all studies described in this report was approved by the local ethics committee from the Federal University of ABC (CEUA/UFABC) under protocol number 009/2013. 

### Cell Culture

Mouse 15P-1 Sertoli cells were purchased from American Type Culture Collection (ATCC^®^). The cells were cultured in DMEM supplemented with 10% FBS at 32°C in a humidified atmosphere containing 95% air and 5% CO_2_. Culture medium was replaced every two days. 

### Crude Venom and Peptide Fraction of *B. jararaca* Venom

*B. jararaca* crude venom (CV) was provided by the Laboratory of Herpetology from Butantan Institute (São Paulo, Brazil), remaining stored at -20°C until use. Protein concentrations of CV were determined using the Bradford reagent (Sigma-Aldrich Corporation, USA) and bovine serum albumin as a standard, according to manufacturer’s instructions. LMWF was obtained from CV with the filtration system (Amicon^^®^^, Merck-Millipore, Germany) in the molecular cutoff membrane of 10 kDa and the filtrate was lyophilized. The fraction obtained was analyzed by SDS-PAGE in 15% polyacrylamide gel, stained with 0.002% of Silver nitrate (AgNO_3_) in formaldehyde 27.75% and subjected to mass spectrometry using Micromass Q-Tof Ultima Mass Spectrometer (MSvision, UK), under positive ionization mode and/or by MALDI-TOF mass spectrometry on an Ettan MALDI-TOF/Prosystem (GE Healthcare/Amersham Biosciences, Sweden) [15]. The yield of this process was determined by the difference between the initial mass of CV and the mass of LWMF obtained at the end of the process.

### Treatment of Animals with LMWF

Mice (n = 12) were anesthetized with Ketamine? and Xylazine? (3:1) at a dose of 174 μg and 11.5 μg per gram of body mass. Initially, they were submitted to a median retro-umbilical longitudinal laparotomy. Right and left testes of animals were exposed in the abdominal cavity, and the peptide fraction was injected directly in the testicular parenchyma of the left testis of each animal (two sites per testis); approximately 10 µL of LMWF (5 µg) diluted in 0.91% w/v NaCl. The vehicle (same volume of 0.91% w/v NaCl) was also administrated in the testicular parenchyma of the right testis of the same animal (control). Each sample was administered using a 0.5-mL syringe and a 30-gauge needle (Ultra-Fine Short Needle, BD, Canada) [14]. Subsequently, the animals were maintained in the animal house for seven days and then were euthanized using CO_2_ asphyxiation. The testes were collected for morphological and morphometric analysis. All treatments were performed in triplicate. 

### Morphological Analyses of Seminiferous Tubules

Testes were fixed in Bouin’s solution (4% formaldehyde with picric acid, v/v) for eight hours, dehydrated in increasing concentrations of alcohol (70% to 95%, v/v) and embedded in Paraplast? (Sigma Chemical Company, USA). Histological slices (4 μm in thickness) were stained with either hematoxylin and eosin or Mallory’s trichrome stain for morphological analysis of the seminiferous epithelium. Periodic acid-Shiff (PAS) with Harris hematoxylin histochemical method was used to determine the stages of the seminiferous epithelium cycle. The sections were examined using a photomicroscope (Axioskop 2, Germany), and the images were captured with a Pixera digital camera system (Pixera Corporation, USA) attached to the photomicroscope and a microcomputer (Intel^®^ Pentium^®^) using the software Adobe Photoshop version 7.0.1 (Adobe Systems, USA). 

Intratesticular administration influence of LMWF in the morphology of the seminiferous tubules was analyzed by semiquantitative histopathological analysis of the seminiferous tubules [14]. Seminiferous tubules of all treatments were classified into four categories: normal tubules (T1); hypospermatogenic tubules (T2); arrested maturation (T3); and tubules containing spermatogonia and Sertoli cells or Sertoli-cell-only tubules (T4). For this purpose, three 4-µm-thick sections of each testis, which were randomly selected, and 100 tubular cross sections were examined from each section. Moreover, intertubular compartment morphology, in particular, Leydig cells, blood vessels, lymph vessels, fibroblasts, macrophages, and mast cells, were analyzed.

### Morphometrical Analyses of Seminiferous Tubules

Stages I, V, VII/VIII and XII of the seminiferous epithelium cycle were analyzed [14,16]. Each stage was identified based on acrosome development and morphology of the nucleus of the spermatids during differentiation [17]. For morphometric analysis, eight circular or nearly circular seminiferous tubules were randomly selected in each studied stage per testis. The images captured were analyzed using the software ImageJ (National Institutes of Health, USA) to assess the diameter of the seminiferous tubules (mm), thickness of the seminiferous epithelium (mm) and diameter of the seminiferous tubule lumen (mm).

The images used for morphometric analysis were also utilized for quantitative analysis. The images were analyzed with the aid of Adobe Photoshop version 7.0.1 (Adobe Systems, USA), with a reticulated grid of 1575 points at 66.7% magnification. Only the nuclei of the cells in the intersection points were counted. The following cell types were counted for each stage (I, V, VII/VIII, and XII) of the seminiferous epithelium cycle: type A spermatogonium, type B spermatogonium, preleptotene spermatocyte, zygotene spermatocyte, meiotic figures, secondary spermatocytes, pachytene spermatocyte, round spermatid and, Sertoli cells. 

The total support capacity of Sertoli cell was performed with the data derived from the quantitative analysis, dividing the total number of germ cells by the total number of Sertoli cells per stage, based on the protocol described in the literature [18]. 

### Claudin-1 Distribution in the Seminiferous Epithelium

Testis sections (4-μm thick) from mice treated with LMWF or control were treated with 1% hydrogen peroxide in methanol (v/v) for 15 min to block endogenous peroxidase activity, permeabilized with 0.1% Triton X-100 (v/v) in PBS for 10 min. Thereafter, sections were incubated with a rabbit anti-claudin-1 antibody (MH25- Zymed/Invitrogen?, lot 50393527, cat. no. 71-7800) diluted (1:250) in 0.05 M Tris-HCl with 1% bovine serum albumin (BSA) overnight. They were washed in PBS three times for 5 min and incubated with the diluted biotinylated anti-rabbit IgG for 30 min, then washed in PBS three times for 5 min and incubated for 30 min with Streptavidin-biotin-peroxidase complex. Immunoreactive sites were revealed using a buffered solution of 3,3’-diaminobenzidine-tetrahydrochloride (DAB) (Dako cytomation?, USA). 

Images were analyzed in a Zeiss Axioskop 2 photomicroscope and captured by Pixera (Pixera Corporation, USA). The images acquired were converted to TIFF format, and analyzed by Adobe Photoshop in Adobe Creative Suite (Version 3.0, Adobe Systems, USA), such as merges of images to assess protein co-localization. Negative controls included the substitution of the primary antibody by normal rabbit IgG (Vector Laboratories) or the omission of the secondary antibody.

### Nitric Oxide Measurement

The nitric oxide levels were evaluated by nitrate and nitrite accumulation in the total extract of the testis protein. For this, four male Swiss mice (weighting 30-35 g) were used. The animals were submitted to perfusion with saline solution 0.9% containing heparin 1:1000. After the testes were collected and these samples were homogenized on ice with a polytron PT MR 3000 homogenizer (Kinematic AG, Switzerland) in a buffer composed of (mM): Tris-HCl 50, EDTA 0.1, EGTA 0.1, 2-mercaptoethanol 12, and phenylmethylsulphonyl fluoride 1 (pH 7.4). An amount of 60 µg of the homogenates was incubated with LMWF (0.1, 1 and 10 µg/mL), sodium nitroprusside (SNP, 1 µM), L-Name (1 µM) or bradykinin (Bk, 100 µM) for the volume of 500 µL, for 1 h. This reaction was stopped with addition of 1% trichloroacetic acid (TCA, 4°C) and, subsequently, these samples were centrifuged for 7 min at 13,000 x *g*. After the reduction of nitrate and nitrite with a saturated chloride vanadium solution (VCl_3_) in HCl 1M, 90ºC, nitrate from the supernatant (30 µL, approximately 60 µg of protein) were injected into the NO analyzer. The nitrate concentration was determined by the curve standard of NaNO_3_ using the Bag program software 2.2. 

### Effects of CV and LMWF on Sertoli Cell Culture

Sertoli cells (15P-1) were seeded onto 96 well plates (SPL life sciences Co., Korea) at 1.0 × 10^4^/well. The cells were treated with CV and LMWF at concentrations of 0.001, 0.01, 0.1, 1.0 and 10 μg/mL for 6, 12, 24 and 48 h of incubation, to determine the dose-response curve. These treatments were intended to check the cytotoxicity of the compounds evaluated in terms of different doses. After, the culture medium was removed and added to 50 μL of MTT (0.5 mg/mL, Sigma-Aldrich, USA) per well. The plate was incubated for 2 h at 37ºC. Then, the medium was removed and added to 150 μL of dimethylsulfoxide (DMSO - Sigma-Aldrich, USA) to dissolve the formazan crystals [19]. After 15 min of homogenization, the cell viability was determined by the absorbance of samples at 590 nm in a microplate reader (Spectramax^®^).

### Statistical Analysis

The software GraphPad Prism (version 4.0; GraphPad Software, Incorporation) was used for statistical analyses. The present study consisted of tests that produce qualitative and quantitative results. Quantitative results were analyzed by one or two-way analysis of variance (ANOVA), followed by Tukey or Bonferroni post-test, respectively. Differences were considered significant when *p* < 0.05. All values were expressed as mean ± standard deviation (SD). For statistical analysis, we used the software GraphPad Prism 4. 

## Results

### Characterization of LMWF

**Figure 1.**
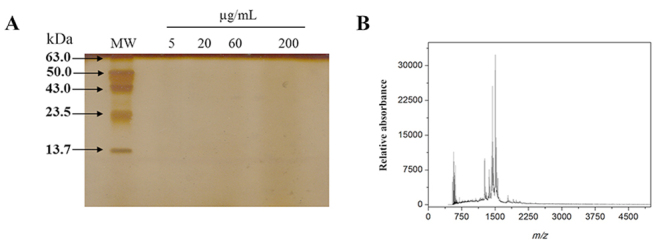
Characterization of the LMWF obtained from *B. jararaca* snake venom. **(** A **)** SDS-PAGE in 15% polyacrylamide gel stained with silver nitrate of LMWF in different concentrations (5, 10, 60 and 200 µg/mL). **(** B **)** Mass spectrometry of LMWF. MW: molecular weight marker; kDa: kilodaltons.

Initially, LMWF obtained from crude venom of *B. jararaca* was analyzed by SDS-PAGE in 15% polyacrylamide gel (silver stained) using different concentrations of the fraction (5, 10, 60 and 200 µg/mL). No protein band corresponding to medium or high molecular mass proteins were identified in the gel (Figure 1A). Besides, electrospray ionization-mass spectrometry (ESI-MS) of the LMWF indicated that mass signal was detected between 440 and 1750 m/z especially, but not above 2250 m/z (Figure 1B). 

### Effect of LMWF on the Structure and Function of Seminiferous Tubules

**Figure 2.**
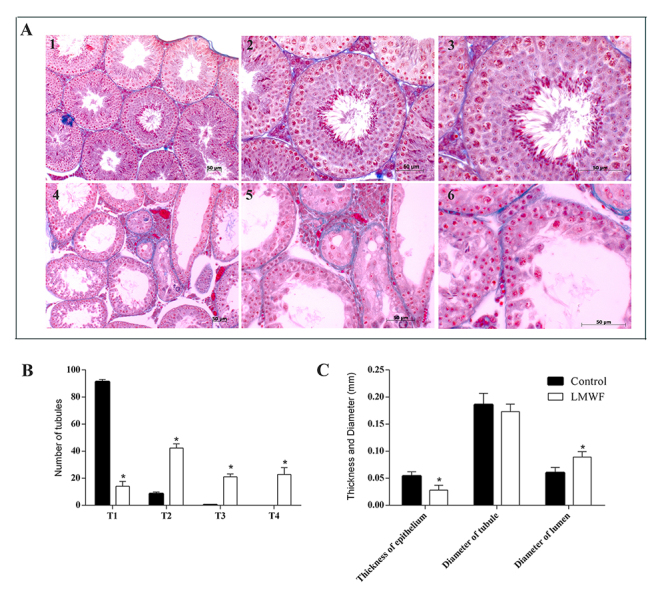
Effects of LMWF in the structure of seminiferous tubules. **(** A **)** Right testis (RT) of control animals treated with 0.91% w/v NaCl (panels 1-3) and the left testis (LT) of mice treated with LMWF diluted in 0.91% w/v NaCl (panels 4-6). Normal structure of tubules in A1-A3. Intense degenerative tubule and ruptures in the epithelium in panels 4-6. **(** B **)** Semiquantitative histological assessment of degree of tubule degeneration. **(** C **)** Morphometric analysis of seminiferous tubules of the RT (control) and LT of animals treated with LMWF. Thickness of the epithelium, diameter and lumen of the seminiferous tubules of control and treated animals were analyzed. Data are presented as the mean ± SD and significant differences as assessed through one-way ANOVA followed by Tukey *post hoc* test (*p < 0.05). Staining: Mallory’s Trichrome. T1: normal tubules; T2: hypospermatogenic tubules; T3: arrested maturation tubules; T4: tubules containing spermatogonia and Sertoli cells or Sertoli-cell-only tubules.

Animals treated with 0.91% NaCl (w/v) displayed normal testicular tissue, particularly regarding the arrangement of germ cells and integrity of the tubule and interstitial compartment (Figure 2A, panels 1-3). In contrast, LMWF showed an absence of elongated spermatids in the seminiferous tubules and discontinuity of the seminiferous epithelium caused by the displacement of germ cells of the adjacent epithelium and ruptures in the seminiferous epithelium (Figure 2A, panels 4-6). LMWF induced tubule degeneration in all four-category scale based on the mean of tubule types present in each group when compared to the control (Figure 2B). Besides, LMWF treatment displayed a reduction in the thickness of the seminiferous epithelium and an increase in the diameter of the seminiferous tubule lumen (Figure 2C), but no alteration was detected in the tubule diameter when compared with vehicle alone. 

**Figure 3.**
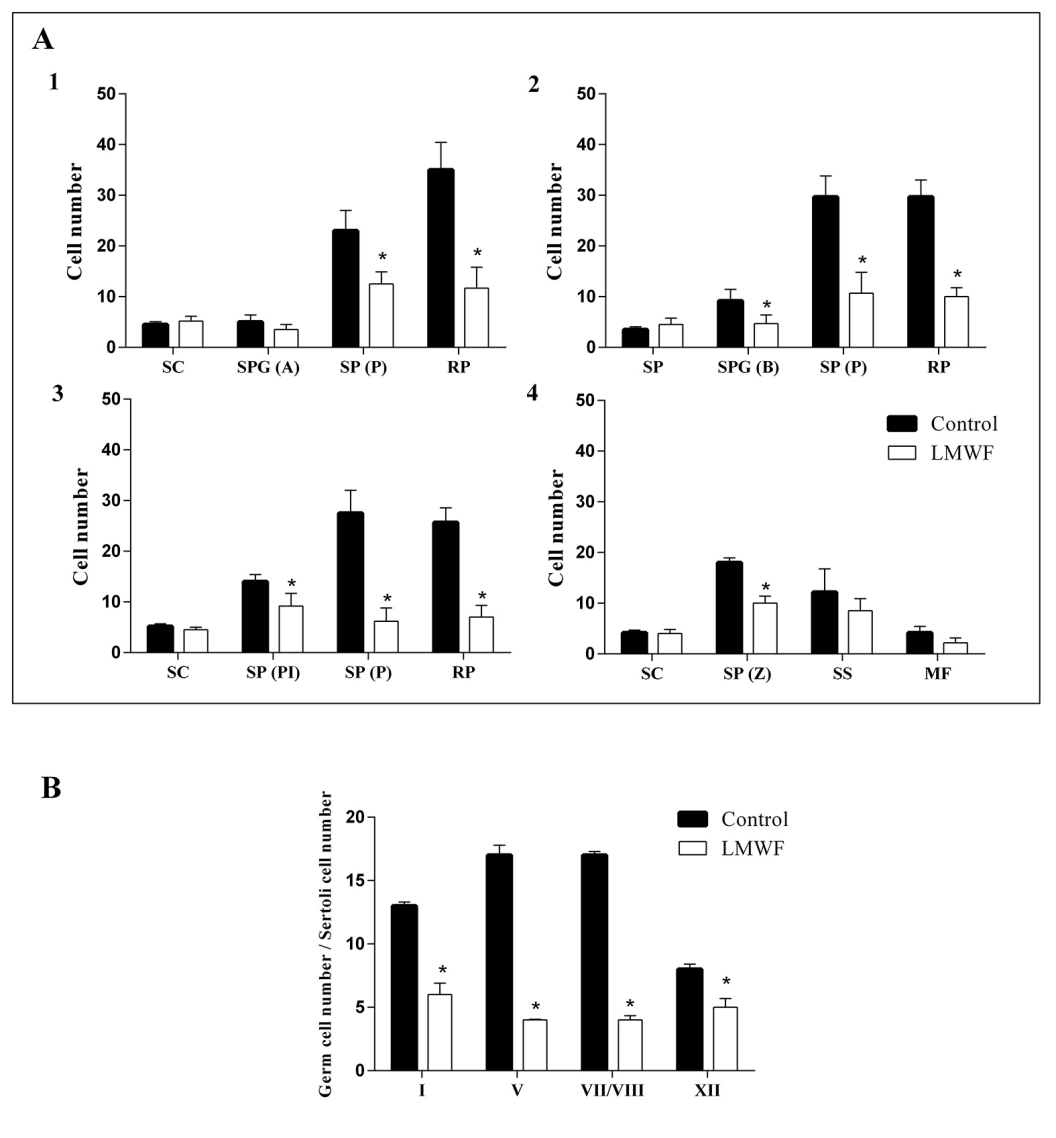
Effects of LMWF in the function of seminiferous epithelium. **(** A **)** Quantitative analysis of germ cell types at I, V, VII/VIII, and XII stages of the seminiferous epithelium cycle (panels 1-4, respectively). **(** B **)** Total support capacity of Sertoli cells during I, V, VII/VIII and XII stages of the seminiferous epithelium cycle. Values are expressed as mean ± standard deviation from three independent experiments in triplicate and analyzed by ANOVA one-way followed by Tukey *post hoc* test. *p <0.05 in relation to control treated with 0.91% w/v NaCl. SC: Sertoli cell; SPG(A): type A spermatogonium; SPG(B): type B spermatogonium; SP(Pl): preleptotene spermatocyte; SP(Z): zygotene spermatocyte; SP(P): pachytene spermatocyte; MF: meiotic figures; SS: secondary spermatocyte; RP: round spermatid.

The spermatocytes in pachytene and round spermatid numbers were significantly reduced in stages I, V and VII/VIII of the seminiferous epithelium cycle in testis treated with LMWF (Figure 3A, panels 1-3). LMWF also decreased the number of preleptotene spermatocytes and zygotene spermatocytes in stages VII/VIII and XII, respectively (Figure 3A, panels 3 and 4). Interestingly, the treatment with LMWF led to a reduction in total support capacity of each Sertoli cell in I, V, VII/VIII and XII stages compared with the control (Figure 3B), but no differences were detected in the number of Sertoli cells.

### Immunohistochemical Analysis of Claudin-1 in the Seminiferous Epithelium after LMWF Treatment

**Figure 4.**
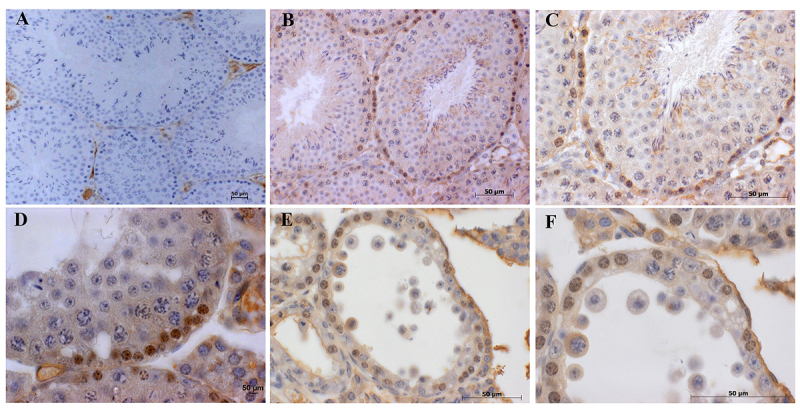
Effects of LMWF on the distribution of claudin-1 in the seminiferous epithelium. **(** A **)** Non-specific staining was detected only in the basal and adluminal compartments of seminiferous epithelium of control sections - negative control. **(** B **-** C) Immunohistochemical staining of RT transverse cross-sections treated with 0.91% w/v NaCl. **(** D **-** F **)** Immunostaining of claudin-1 following treatment with LMWF demonstrated no difference in the distribution of claudin-1 when compared to the control group treated with 0.91% w/v NaCl. Hematoxylin was used for counterstaining.

Claudin-1 appears as a reddish-brown precipitate in the basal compartment of normal seminiferous tubules in the control group (Figure 4B and 4C). Immunoreactive claudin-1 precipitate is formed in the basal and adluminal compartments of each tubule in every stage of the germinal epithelium cycle. Moreover, immunoreactive specificity could be observed in the nucleus of premeiotic germ cells, but not in pachytene spermatocytes, secondary spermatocytes, or round spermatids. Likewise, the distribution of claudin-1 following LMWF (Figure 4D, 4E and 4F) treatment was the same as that of the seminiferous epithelium in untreated mice, especially in the basal compartment. Non-specific staining was only detected in the seminiferous epithelium of control sections, illustrating that the immunoreactivity is specific for claudin-1 (Figure 4A). 

### Nitric Oxide Measurement

**Figure 5.**
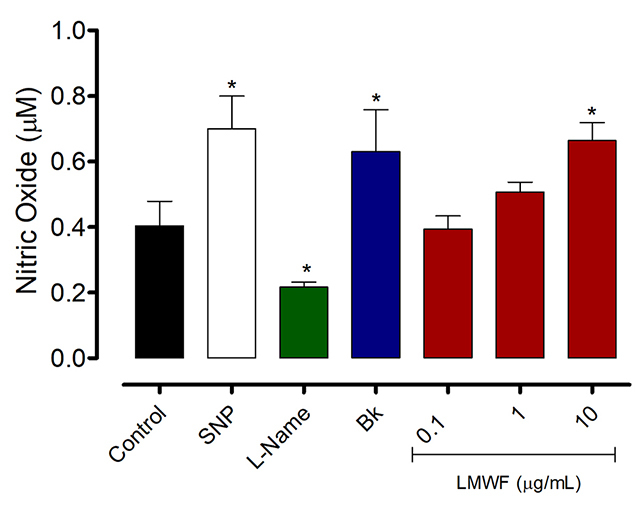
Effects of LMWF on the nitric oxide accumulation in the total extract of the testis protein. Homogenates samples (60 µg of protein) were incubated for 1 h with the LMWF (0,1, 1 and 10 µg/mL), sodium nitroprusside (SNP, 1 mM), L-NAME (1 mM) or bradykinin (Bk, 100 µM). Values are expressed as mean ± standard deviation from three independent experiments in triplicate and analyzed by ANOVA one-way followed by Tukey *post hoc* test. *p < 0.05 to control.

LMWF at 10 μg/mL increased the NO levels in the total extract of the testis protein after 1 h of incubation as well as sodium nitroprusside and bradykinin when compared to the controls. In contrast, L-NAME reduced the NO levels as expected (Figure 5). 

### Toxicity of CV and LMWF on Sertoli Cell Culture

**Figure 6.**
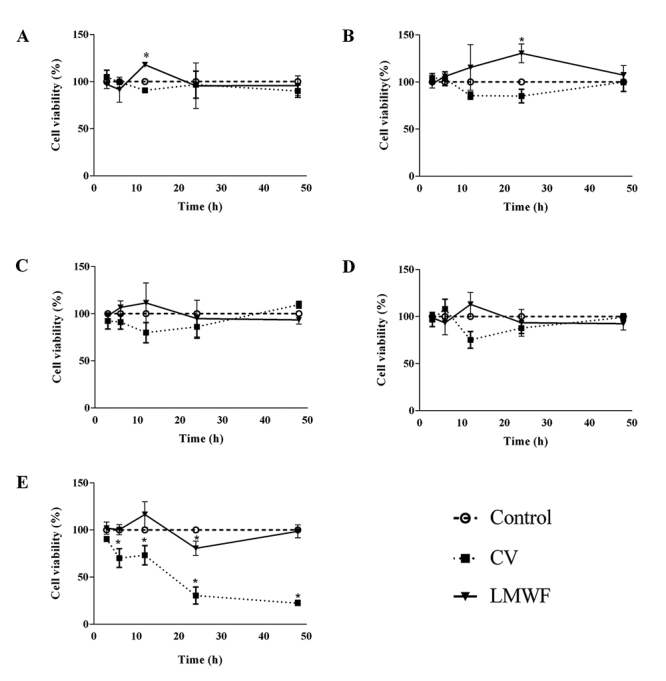
Toxicity of crude venom (CV) and LMWF of *B. jararaca* venom on the Sertoli cell culture (15P-1). Cells treated with either CV or LMWF in the following concentrations **(** A **)** 0.001, **(** B **)** 0.01, **(** C **)** 0.1, **(** D **)** 1.0 and **(** E **)** 10 μg/mL for 2, 6, 12, 24 and 48 h. Values are expressed as mean ± standard deviation from three independent experiments in triplicate and analyzed by ANOVA one-way followed by Tukey *post hoc* test. *p < 0.05 to control without treatment.

LMWF was not cytotoxic in all tested conditions, compared with the control group (Figure 6). Interestingly, the cells treated with low concentrations of LMWF (0.001 μg/mL at 12h and 0.01 μg/mL at 24h) increased cell viability when compared to the control and CV groups. On the other hand, CV showed cytotoxicity at 10 μg/mL from 6 to 48h of treatment. 

## Discussion

Herein, we investigated the toxicological effects of LMWF obtained from *B. jararaca* snake venom, containing a range of < 10 kDa compounds, on the testis of adult mice, an important organ of the male reproductive system. Interestingly, LMWF inhibited the spermatozoa production; principally in the spermiogenesis stage without altering claudin-1 distribution in the basal compartment of seminiferous epithelium. Besides, the changes in the structure and function of the seminiferous epithelium induced by LMWF could be explained by the NO increase that opens complex junction and releases the cells of adluminal compartment to the lumen of seminiferous tubules. Envenomation induced by *Bothrops* species are well-characterized [9,10,20,21], but as far as we know, there is no study about a possible action of *B. jararaca* snake venom on the male reproductive system of victims. Therefore, the present results may provide a better knowledge of the clinical aspects of envenomation and the identification and implementation of a therapeutic strategy. 

LMWF comprises a series of bioactive peptides, especially bradykinin potentiating peptides (BPPs), that in general possess hypotensive action [22-26]. BPPs were the first natural angiotensin-converting enzyme (ACE) inhibitors that inhibit hypotensive peptide (bradykinin) degradation and vasoconstrictor peptide (angiotensin-II) formation [27]. Our group has been demonstrated that BPP-10c, and not captopril, modifies spermatogenesis by causing hyperplasia of round spermatids in stages I, V, and VII/VIII of the spermatogenic cycle, although both are ACE inhibitors [13]. In the present study, we demonstrated for the first time that LMWF induced significant alteration in the structure and function of the seminiferous epithelium. In addition, it decreases the number of round spermatid in stages I, V and VII/VIII of the seminiferous epithelium, indicating a possible inhibition of spermatozoa production, principally in the spermiogenesis stage. 

Spermatogenesis takes place in the seminiferous epithelium of the mammalian testis where germ cells residing in the basal compartment must traverse the blood-testis barrier and enter the adluminal compartment for further development into round, elongated spermatids [28]. Nitric oxide (NO) regulates the cell junction dynamics in the seminiferous epithelium via the NO/soluble guanylate cyclase/cGMP protein kinase G/b-catenin signaling pathway [29], which contributes to the control of migration of developing germ cells of the basal compartment to the adluminal compartment [28,30-32]. Interestingly, LMWF treatment reduced the number of cells in the adluminal compartment and, consequently, diminished the thickness of the seminiferous epithelium and total support capacity of each Sertoli cell. LMWF also increased the NO levels in the total extract of the testis protein. Interestingly, these results suggest that the NO increase by LMWF could explain the rupture of seminiferous epithelium due to action of NO that opens complex junctions and releases the cells from the adluminal compartment to the lumen of seminiferous tubule. This hypothesis could explain the decrease in total support capacity of each Sertoli cell in I, V, VII/VIII and XII stages of the spermatogenic cycle in animals treated with LMWF. 

Claudin-1 is e expressed in the mammalian testis [33] and is found in the basal and adluminal compartments of the seminiferous epithelium [13]. Besides, it interacts with multiple proteins and is intimately involved in signal transduction in the tight junctions [34]. In our study, the localization of claudin-1 in the seminiferous epithelium was examined to assess its possible changes in the BTB during the LMWF-induced spermatogenesis damage. No alterations were shown in the distribution of claudin-1 in animals treated with LMWF, especially in the basal compartment, suggesting that the peptide did not alter BTB integrity. 

In mammalian testes, Sertoli cells are the primary supportive cells of the seminiferous epithelium and play a key role in triggering and regulating spermatogenesis [35]. Sertoli cells also act as phagocytes, consuming residual cytoplasm from the spermiogenesis process [17,18]. Failure in the function and development of Sertoli cells results in the loss of their ability to support germ cell survival and development and may lead to spermatogenesis disturbance [36]. For this reason, an *in vitro* culture model of 15P-1 Sertoli cells was employed to investigate the toxicological effects of LMWF in this cell type of the seminiferous epithelium. The 15P-1 Sertoli cell line was characterized originally as Sertoli cells based on their morphology and steroid metabolism [37-39]. Since its generation, this cell line has been used in numerous studies with results similar to those obtained using primary cultures of Sertoli cells [40-45]. There are no reports about the LMWF effects on the cell viability in cultured Sertoli cells, and it was not cytotoxic in concentrations and time tested in the present study. Similarly, LMWF did not demonstrate a change in cultivated hippocampal cells [15] and human neuroblastoma (SH-SY5Y) cell viability [46]. It is important to emphasize that the LMWF obtained from *B. jararaca* was characterized by different methods to ensure that the purified fraction had only peptide components with less than 10 kDa and the absence of high molecular weight proteins. On the other hand, CV showed cytotoxicity at 10 μg/mL from 6 to 48 h of treatment as expected, because *B. jararaca* venom is composed of many toxic compounds responsible for tissue injury and hemorrhage [47]; among them, many are cytotoxic in different cell types [15,46]. 

## Conclusion

LMWF from *B. jararaca* snake venom changed the structure and function of the seminiferous epithelium in mice, indicating a possible inhibition of spermatozoa production; principally in the spermiogenesis stage without altering claudin-1 distribution - especially in the basal compartment. Moreover, NO increase by LMWF could explain the decrease of germinative cells in seminiferous epithelium due to the action of NO that opens complex junctions and releases the cells of adluminal compartment to the lumen of seminiferous tubule. Further analyses will contribute to a better understanding of the possible action of *B. jararaca* snake venom on the male reproductive system of affected individuals, thereby providing new insight into the biological features of snake venom and therapeutic strategies against envenomation.

### Abbreviations

ACE: angiotensin-converting enzyme; BPPs: bradykinin potentiating peptides; BTB: blood-testis barrier; CV: crude venom; ESI-MS: electrospray ionization-mass spectrometry; i.t.: intratesticular; LMWF: low molecular weight fraction; LT: left testis; NO: nitric oxide; PAS: periodic acid-Shiff; RT: right testis; SD: standard deviation.

## References

[B1] World Health Organization (2014). Encycl. Qual. Life Well-Being Res.

[B2] Fox JW, Serrano SMT (2008). Exploring snake venom proteomes: Multifaceted analyses for complex toxin mixtures. Proteomics.

[B3] Hayashi MAF, Camargo ACM (2005). The Bradykinin-potentiating peptides from venom gland and brain of Bothrops jararaca contain highly site specific inhibitors of the somatic angiotensin-converting enzyme. Toxicon.

[B4] Ehlers MRW, Fox EA, Strydom DJ, Riordan JF (1989). Molecular cloning of human testicular angiotensin-converting enzyme: The testis isozyme is identical to the C-terminal half of endothelial angiotensin-converting enzyme. Proc Natl Acad Sci U S A.

[B5] Cushman DW, Ondetti MA (1999). Design of angiotensin converting enzyme inhibitors. Nat. Med.

[B6] Hayashi MAF, Murbach AF, Ianzer D, Portaro FCV, Prezoto BC, Fernandes BL (2003). The C-type natriuretic peptide precursor of snake brain contains highly specific inhibitors of the angiotensin-converting enzyme. J Neurochem.

[B7] Silva CA, Ianzer DA, Portaro FCV, Konno K, Faria M, Fernandes BL (2008). Characterization of urinary metabolites from four synthetic bradykinin potentiating peptides (BPPs) in mice. Toxicon.

[B8] Luna KPO, da Silva MB, Pereira VRA (2011). Clinical and immunological aspects of envenomations by Bothrops snakes. J Venom Anim Toxins incl Trop Dis.

[B9] Santoro ML, Sano-Martins IS, Fan HW, Cardoso JLC, Theakston RDG, Warrell DA (2008). Haematological evaluation of patients bitten by the jararaca, Bothrops jararaca, in Brazil. Toxicon.

[B10] Gonçalves LRC, Mariano M (2000). Local haemorrhage induced by Bothrops jararaca venom: Relationship to neurogenic inflammation. Mediators Inflamm.

[B11] Kumar JR, Basavarajappa BS, Arancio O, Aranha I, Gangadhara NS, Yajurvedi HN (2008). Isolation and characterization of “Reprotoxin”, a novel protein complex from Daboia russelii snake venom. Biochimie.

[B12] Fernandes FH, Bustos-Obregon E, Matias R, Dourado DM (2018). Crotalus durissus sp. rattlesnake venom induces toxic injury in mouse sperm. Toxicon.

[B13] Gilio JM, Portaro FC, Borella MI, Lameu C, Camargo AC, Alberto-Silva C (2013). A bradykinin-potentiating peptide (BPP-10c) from Bothrops jararaca induces changes in seminiferous tubules. J Venom Anim Toxins incl Trop Dis.

[B14] Alberto-Silva C, Gilio JM, Portaro FCV, Querobino SM, Camargo ACM (2015). Angiotensin-converting enzyme inhibitors of Bothrops jararaca snake venom affect the structure of mice seminiferous epithelium. J Venom Anim Toxins incl Trop Dis.

[B15] Querobino SM, Carrettiero DC, Costa MS, Alberto-Silva C (2017). Neuroprotective property of low molecular weight fraction from B. jararaca snake venom in H2O2-induced cytotoxicity in cultured hippocampal cells. Toxicon.

[B16] Kaneto M, Kanamori S, Hishikawa A, Kishi K (1999). Epididymal sperm motion as a parameter of male reproductive toxicity: Sperm motion, fertility, and histopathology in ethinylestradiol-treated rats. Reprod Toxicol.

[B17] Russell LD, Ettlin RA, Hikim APS, Clegg ED (1993). Histological and histopathological evaluation of the testis. Int J Androl.

[B18] Russell LD, Peterson RN (1984). Determination of the elongate spermatid-Sertoli cell ratio in various mammals. J Reprod Fertil.

[B19] Stockert JC, Blázquez-Castro A, Cañete M, Horobin RW, Villanueva Á (2012). MTT assay for cell viability: Intracellular localization of the formazan product is in lipid droplets. Acta Histochem.

[B20] Otero-Patiño R (2009). Epidemiological, clinical and therapeutic aspects of Bothrops asper bites. Toxicon.

[B21] Del Brutto OH, Del Brutto VJ (2012). Neurological complications of venomous snake bites: A review. Acta Neurol Scand.

[B22] Ferreira SH (1965). A bradykinin-potentiating factor (Bpf) present in the venom of Bothrops jararaca. Br J Pharmacol Chemother.

[B23] Ferreira SH, Bartelt DC, Greene LJ (1970). Isolation of bradykinin-potentiating peptides from Bothrops jararaca venom. Biochemistry.

[B24] Ferreira SH, Greene LJ, Alabaster VA, Bakhle YS, Vane JR (1970). Activity of various fractions of bradykinin potentiating factor against angiotensin I converting enzyme. Nature.

[B25] Ianzer D, Konno K, Marques-Porto R, Vieira Portaro FC, Stöcklin R, Martins De Camargo AC (2004). Identification of five new bradykinin potentiating peptides (BPPs) from Bothrops jararaca crude venom by using electrospray ionization tandem mass spectrometry after a two-step liquid chromatography. Peptides.

[B26] Sciani JM, Pimenta DC (2017). The modular nature of bradykinin-potentiating peptides isolated from snake venoms. J Venom Anim Toxins incl Trop Dis.

[B27] Camargo ACM, Ianzer D, Guerreiro JR, Serrano SMT (2012). Bradykinin-potentiating peptides: beyond captopril. Toxicon.

[B28] Su W, Mruk DD, Cheng CY (2013). Regulation of actin dynamics and protein trafficking during spermatogenesis--insights into a complex process. Crit Rev Biochem Mol Biol.

[B29] Da Ni F, Hao SL, Yang WX (2019). Multiple signaling pathways in Sertoli cells: recent findings in spermatogenesis. Cell Death Dis.

[B30] Cheng CY, Mruk DD (2002). Cell junction dynamics in the testis: Sertoli-germ cell interactions and male contraceptive development. Physiol Rev.

[B31] Lui WY, Cheng CY (2007). Regulation of cell junction dynamics by cytokines in the testis-a molecular and biochemical perspective. Cytokine Growth Factor Rev.

[B32] Lui W, Lee WM, Cheng CY (2003). Sertoli-germ cell adherens junction dynamics in the testis are regulated by RhoB GTPase via the ROCK/LIMK signaling pathway. Biol Reprod.

[B33] Gye MC (2003). Expression of claudin-1 in mouse testis. Arch Androl.

[B34] Mitic LL, Van Itallie CM, Anderson JM (2000). Molecular physiology and pathophysiology of tight junctions I. Tight junction structure and function: lessons from mutant animals and proteins. Am J Physiol Gastrointest Liver Physiol.

[B35] De Kretser DM, Loveland KL, Meinhardt A, Simorangkir D, Wreford N (1998). Spermatogenesis. Hum Reprod.

[B36] Gao Y, Mruk DD, Cheng CY (2015). Sertoli cells are the target of environmental toxicants in the testis - a mechanistic and therapeutic insight. Expert Opin Ther Targets.

[B37] Vidal F, Lopez P, Lopez-Fernandez LA, Ranc F, Scimeca JC, Cuzin F (2019). Expression of Concern: Gene trap analysis of germ cell signaling to sertoli cells: NGF-TrkA mediated induction of Fra1 and Fos by post-meiotic germ cells. J Cell Sci.

[B38] Vincent S, Segretain D, Nishikawa S, Nishikawa SI, Sage J, Cuzin F (1998). Stage-specific expression of the Kit receptor and its ligand (KL) during male gametogenesis in the mouse: A Kit-KL interaction critical for meiosis. Development.

[B39] Rassoulzadegan M, Paquis-Flucklinger V, Bertino B, Sage J, Jasin M, Miyagawa K (1993). Transmeiotic differentiation of male germ cells in culture. Cell.

[B40] Lin J, Zhu J, Li X, Li S, Lan Z, Ko J (2014). Expression of genomic functional estrogen receptor 1 in mouse sertoli cells. Reprod Sci.

[B41] Wang L, Hao J, Hu J, Pu J, Lü Z, Zhao L (2012). Protective effects of ginsenosides against Bisphenol A-induced cytotoxicity in 15P-1 Sertoli cells via extracellular signal-regulated kinase 1/2 signalling and antioxidant mechanisms. Basic Clin Pharmacol Toxicol.

[B42] Yang Q, Hao J, Chen M, Li G (2014). Dermatopontin is a novel regulator of the CdCl2-induced decrease in claudin-11 expression. Toxicol In Vitro.

[B43] Ghouili F, Roumaud P, Martin LJ (2018). Gja1 expression is regulated by cooperation between SOX8/SOX9 and cJUN transcription factors in TM4 and 15P-1 Sertoli cell lines. Mol Reprod Dev.

[B44] Hu Z, Dandekar D, O’Shaughnessy PJ, De Gendt K, Verhoeven G, Wilkinson MF (2010). Androgen-induced Rhox homeobox genes modulate the expression of AR-regulated genes. Mol Endocrinol.

[B45] Yu J, Sun J, Fan Y, Su J, Xie J, Wu Y (2019). Exposure to Pb and Cd alters MCT4/CD147 expression and MCT4/CD147-dependent lactate transport in mice Sertoli cells cultured in vitro. Toxicol In Vitro.

[B46] Querobino SM, Ribeiro CAJ, Alberto-Silva C (2018). Bradykinin-potentiating PEPTIDE-10C, an argininosuccinate synthetase activator, protects against H2O2-induced oxidative stress in SH-SY5Y neuroblastoma cells. Peptides.

[B47] de souza LL, Stransky S, Guerra-duarte C, Flor-Sá A, Schneider FS, Kalapothakis E (2015). Determination of toxic activities in Bothrops spp. snake venoms using animal-free approaches: Correlation between in vitro versus in vivo assays. Toxicol Sci.

